# Exploring mitochondrial biomarkers for Friedreich's ataxia: a multifaceted approach

**DOI:** 10.1007/s00415-024-12223-5

**Published:** 2024-03-23

**Authors:** Lucie Stovickova, Hana Hansikova, Jitka Hanzalova, Zuzana Musova, Valerij Semjonov, Pavel Stovicek, Haris Hadzic, Ludmila Novotna, Martin Simcik, Pavel Strnad, Anastaziia Serbina, Simona Karamazovova, Jaroslava Schwabova Paulasova, Martin Vyhnalek, Pavel Krsek, Alena Zumrova

**Affiliations:** 1 Department of Paediatric Neurology, Second Faculty of Medicine, Charles University, Motol University Hospital, V Uvalu 84, 15006 Prague 5, Czech Republic; 2 Department of Paediatrics and Inherited Metabolic Disorders, First Medical Faculty, Charles University and General University Hospital in Prague, Prague 2, Czech Republic; 3 Department of Immunology, Second Faculty of Medicine, Charles University, Motol University Hospital, Prague 5, Czech Republic; 4 Department of Biology and Medical Genetics, Second Faculty of Medicine, Charles University, Motol University Hospital, Prague 5, Czech Republic; 5 Second Faculty of Medicine, Charles University, Prague 5, Czech Republic; 6 Department of Neurology, Second Faculty of Medicine, Charles University, Motol University Hospital, Prague 5, Czech Republic; 7 Centre of Hereditary Ataxias, Second Faculty of Medicine, An Official EFACTS Site, a Member of European Reference Network for Rare Neurological Diseases (ERN-RND), Charles University, Motol University Hospital, Prague 5, Czech Republic; 8 Department of Paediatrics, Second Faculty of Medicine, Charles University and Motol University Hospital, Prague, Czech Republic; 9 Prague, Czech Republic

**Keywords:** Friedreich's ataxia, Mitochondrial dysfunction, Complex IV (COX), Complex I (NQR), Complex II (SQR), Ubiquinone (Coenzyme Q10), Neurofilament light chain (NFL), Biomarkers, Disease monitoring, Therapeutic monitoring

## Abstract

This study presents an in-depth analysis of mitochondrial enzyme activities in Friedreich's ataxia (FA) patients, focusing on the Electron Transport Chain complexes I, II, and IV, the Krebs Cycle enzyme Citrate Synthase, and Coenzyme Q10 levels. It examines a cohort of 34 FA patients, comparing their mitochondrial enzyme activities and clinical parameters, including disease duration and cardiac markers, with those of 17 healthy controls. The findings reveal marked reductions in complexes II and, specifically, IV, highlighting mitochondrial impairment in FA. Additionally, elevated Neurofilament Light Chain levels and cardiomarkers were observed in FA patients. This research enhances our understanding of FA pathophysiology and suggests potential biomarkers for monitoring disease progression. The study underscores the need for further clinical trials to validate these findings, emphasizing the critical role of mitochondrial dysfunction in FA assessment and treatment.

**Supplementary Information:**

The online version contains supplementary material available at 10.1007/s00415-024-12223-5.

## Introduction

Friedreich's ataxia (FA) is an autosomal recessive genetic disease caused mainly by GAA repeats in the FXN gene, with the shorter GAA1 allele more closely linked to disease progression. It leads to frataxin deficiency impacting mitochondrial functions. FA is a multi-systemic disorder characterized by a range of clinical symptoms including neurological manifestations, such as ataxia and dysarthria, cardiomyopathy, skeletal deformities like scoliosis and pes cavus, diabetes mellitus, and additional complications [1–6].

The oxidative phosphorylation system (OXPHOS), key for cellular energy, includes five main complexes: NADH-coenzyme Q10 oxidoreductase (NQR, Complex I), succinate-coenzyme Q10 oxidoreductase (SQR, Complex II), ubiquinol-cytochrome c oxidoreductase (QCCR, Complex III), cytochrome c oxidase (COX, Complex IV), and ATP synthase (Complex V) [7–10]. Current understanding of FA is linked to OXPHOS dysfunction, which is associated with frataxin deficiency impacting iron–sulfur (Fe–S) cluster formation and interfering with some of the OXPHOS complexes [11–21]. Coenzyme Q10 (Q10), essential for electron transfer in OXPHOS [8, 9], has been explored as a treatment option. However, its efficacy in FA has been found to be limited [22, 23]. The link between OXPHOS impairment and cardiomyopathies, including FA, was also reported [24–27].

Frataxin is involved in Fe–S cluster production and heme formation, its deficiency correlates with increased production of reactive oxygen species (ROS), leading to oxidative stress and cellular damage. Elevated ROS levels can contribute to a range of harmful cellular effects, such as inflammasome activation, apoptosis, and ferroptosis [28, 29].

Recent advancements in FA treatment have seen progress including innovative approaches like gene editing and epigenetics. A notable development is omaveloxolone, a nuclear factor erythroid 2-related factor 2 (Nrf2) activator, which became the first FDA-approved FA treatment in February 2023 [30]. Omaveloxolone influences the Nrf2 pathway, which helps improve mitochondria and cell resilience against ROS and cascade of following bioprocesses [31, 32].

**Fig. 1:**
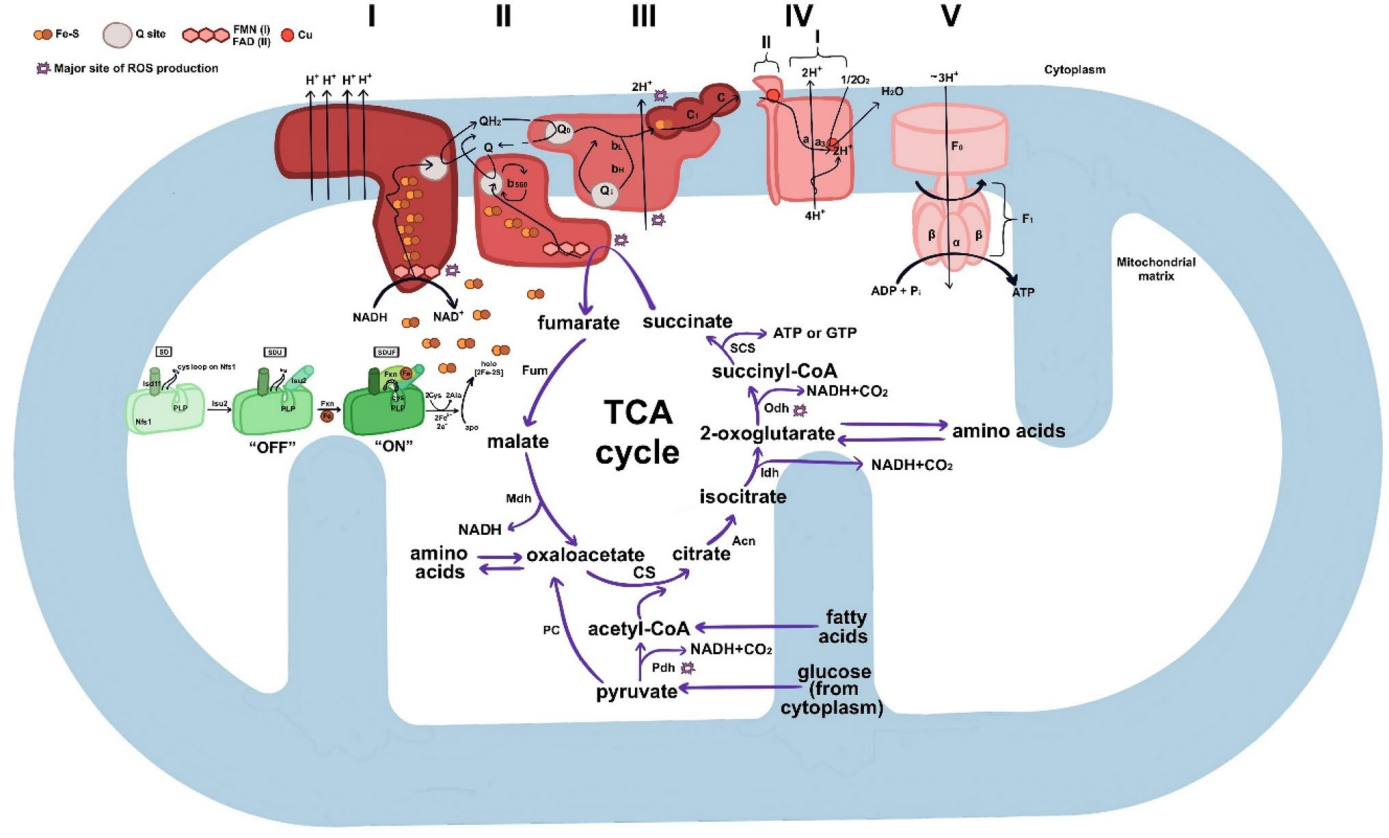
Integrated Schematic of Frataxin’s Multi-Faceted Role in Mitochondrial Bioprocesses and its Implications in Friedreich's Ataxia. The information depicted in this figure is adapted and synthesized from publications presented by Tsai et al. 2010 and Mailloux et al. 2013 and 2015. Figure offers a comprehensive schematic that illustrates the pivotal role of frataxin in Iron–Sulfur (Fe–S) cluster synthesis and its subsequent interactions with the TCA cycle, ETC, and OXPHOS within the mitochondrial matrix. This understanding holds particular significance for disorders like FA, where compromised Fe–S cluster synthesis plays a detrimental role. Deficiencies in frataxin and Fe–S clusters can severely hamper the mitochondrial bioprocesses, including efficient electron transfer and ATP production, emphasizing the need to understand these relationships in both normal and pathological conditions. Left Section (Green): Frataxin’s Role in Fe–S Cluster Biosynthesis [16]. The left portion of the figure highlights the mechanics of Fe–S cluster biosynthesis facilitated by frataxin. It interacts with a multi-protein complex comprised of Nfs1, Isd11, and Isu2, activating the Fe–S cluster biosynthetic pathway from its “off” state to “on state”. Upper Section (Red): Electron Transport Chain (ETC) and OXPHOS [9, 21]. The upper section of the figure elucidates how NADH and succinate, byproducts of the TCA cycle, are oxidized by NQR and SQR, both of which rely on Fe–S clusters for effective electron transfer. NQR needs 7–8 Fe–S clusters for the systematic transfer of electrons from NADH to Q10, converting it into ubiquinol (QH_2_). SQR uses 3 Fe–S clusters to convert succinate and facilitate the transfer of electrons to Q10 (this process links ETC with TCA cycle). These processes are intrinsically linked with proton pumping, setting up a proton motive force that Complex V (ATP Synthase) uses to synthesize ATP, a process known as "coupled" respiration or OXPHOS. Lower Section (Purple): TCA Cycle Integration [8]. The lower part of the figure depicts the initiation of the TCA cycle by citrate synthase (CS). CS catalyzes the formation of citrate from acetyl-CoA and oxaloacetate, which is critical for generating NADH, which then feeds into the OXPHOS pathway, creating an integration of nutrient metabolism (fatty acids, amino acids, and glucose) with cellular energy production (ADP to ATP). By providing an integrated view, Fig. 1 serves as a foundational reference for understanding the complex interrelationships among these vital mitochondrial processes analyzed in this study, and how their disruption can interfere with surrounding processes. a and a_1_—two heme groups, *Acn* Aconitase, *ADP* Adenosin Diphosphate, *Ala* Alanine (assuming this is the amino acid), *ATP* Adenosine Triphosphate, *b*_*560*_ Cytochrome b560, *b*_*H*_ Cytochrome b High potential, *b*_*L*_ Cytochrome b Low potential, *C* Cytochrome c, *CS* Citrate Synthase, *Cu* Copper, *Cys* Cysteine, *e*^*−*^ Electron, *F*_*0*_* and F*_*1*_ Subunits of ATP Synthase, *FAD* Flavin Adenine Dinucleotide, *Fe–S* Iron–Sulfur Cluster, *Fe*^*3+*^ Ferric Ion, *FMN* Flavin Mononucleotide, *Fum* Fumarase, *Fxn* Frataxin, *GTP* Guanosine 5’-Triphosphate, *H*^*+*^ Proton, *Idh* Isocitrate Dehydrogenase, *I* *(Complex I)* NADH:Ubiquinone Oxidoreductase (NQR), *II* *(Complex II)* Succinate:Quinone Oxidoreductase (SQR), III* (Complex III)* Ubiquinol:Cytochrome c Oxidoreductase, *IV* *(Complex IV)* Cytochrome c Oxidase (COX) with subunits I (on the right) and II (on the left), *Isd11* Nfs1 interacting protein, *Isu2* Scaffold Protein for Iron–Sulfur (Fe–S) Cluster Biogenesis, *Mdh* Malate Dehydrogenase, *Nfs1* Cysteine Desulfurase, *Odh* 2-Oxoglutarate Dehydrogenase, *Pdh* Pyruvate Dehydrogenase, *PC* Pyruvate Carboxylase, *P*_*i*_ Inorganic phosphate, *PLP* pyridoxal 5′ phosphate, *Q* Quinone (Q10), *QH*_*2*_ Reduced Quinone, *ROS* Reactive Oxygen Species, *SCS* Succinyl-CoA Synthase, *SD* Nfs1 and Isd11 Protein Complex, *SDU* Nfs1, Isd11, and Isu2 Protein Complex, *SDUF* Nfs1, Isd11, Isu2, and Frataxin Protein Complex, *UCP* Uncoupling Protein, *V* *(Complex V)* ATP Synthase

Citrate synthase (CS) is another key mitochondrial enzyme that affects energy production in the Krebs cycle (TCA) and consequently to the electron transport chain (ETC) [8]. See Fig. 1 for schematic overview of all these above-mentioned mitochondrial processes.

A decrease in CS activity has been associated with impaired ATP synthesis and increased susceptibility to neurodegenerative diseases [18, 33]. A study [19] focused on FA reported decreased CS and the OXPHOS system activity in calf muscles. Fibroblasts from skin of 5 FA patients showed increased CS activity [34]. Another study [13], focused on an acute frataxin knockdown in induced pluripotent stem cell-derived cardiomyocytes (iCMs), provides strong evidence of the interplay between frataxin knockdown and mitochondrial dysfunction, significantly affecting the ETC and TCA cycle enzymes, including NQR and SQR subunits, CS, and others. CS serves as a robust marker of mitochondrial mass and to some extent, it indicates a presence of intact mitochondria [35, 36].

Neurofilaments, essential for neuron structure and function, are affected by mitochondrial dysfunction in neurodegenerative diseases, causing elevated levels that suggest axonal damage. In FA, studies show increased serum levels of neurofilament light chain (NFL) and phosphorylated heavy chain (pNFH), indicating their potential as biomarkers of neurodegeneration [37–40]. However, these elevations might also signify axonal remodeling in FA [39, 40]. Notably, NFL levels decrease with age in FA patients, suggesting age as a key factor in NFL dynamics [39, 40].

Our study focuses on assessing mitochondrial markers in FA patients compared to healthy controls, specifically analyzing NQR, SQR, COX, CS, and Q10. We aim to understand both the variations in these markers and their interrelations. Additionally, we explore the link between the activities of respiratory chain enzymes and various clinical and demographic variables in FA, thereby connecting mitochondrial dysfunction to the FA clinical severity, incl. NFL levels. Furthermore, our investigation delves into the relationship between mitochondrial markers and cardiac health in FA, examining the correlation between respiratory chain enzyme activities and cardiac markers. Building on the understanding of FA pathogenesis, this study aims to evaluate a set of potentially clinically relevant biomarkers in FA patients.

## Methods

### Participants and study design

This observational research project involved 41 Czech FA patients registered for baseline examination in the European Friedreich Ataxia Consortium for Translational Studies (EFACTS) registry (clinical trial.gov NCT02069509) from 07/2021 to 06/2022. Patient selection criteria included genetically confirmed FA, signed informed consent as approved by the ethics committee of Motol University Hospital and the Second Faculty of Medicine, Charles University (Approval No. EK-1340.19/20, dated 11/4/2020), and the ability to meet specific laboratory requirements for sample collection. Due to logistical constraints, not all registered patients could participate, resulting in 34 patients being included for Q10 level assessment and 28 for mitochondrial enzyme activity analysis. The selection criteria for the 17 healthy controls primarily focused on the absence of major neurodegenerative or neuro-inflammatory disorders, as verified by medical history and examinations. The control group was chosen to approximate the FA patient group's demographics. No formal power analysis was conducted, considering the study's observational nature. The sample size was determined by logistical and resource limitations and was deemed adequate for preliminary exploration of mitochondrial biomarkers.

Clinical data for the 34 FA patients were sourced from the EFACTS registry. This included current medications, with a focus on FA treatment and Q10 supplementation to determine potential biases in results, especially in the Q10 levels. Other key clinical and demographic variables entered to the EFACTS registry were used. Those include age at examination, gender, age at disease onset, and counted disease duration (age at examination minus age at disease onset). Genetic factors, namely the number of GAA1 / GAA2 repeats in the shorter / longer alleles and the presence of point mutations, were quantified at the official EFACTS laboratory in Milan, Italy. Mobility metrics were obtained also from EFACTS registry, including the disability stage (rated on a scale from 1 to 7, 1 means “no functional handicap but signs at examination” and 7 means “confined to bed”) and the severity index (disease stage/disease duration), the use of permanent walking aid and/or wheelchair. Clinical measures also included total scores on the Montreal Cognitive Assessment (MoCA), the Friedreich's Ataxia Rating Scale – Activities of Daily Living subset (ADL) and the Scale for the Assessment and Rating of Ataxia (SARA). The average annual worsening of ADL (ADL/disease duration) and SARA scores (SARA/disease duration) was calculated, considering the partial non-linearity of both scales. Additionally, the serum neurofilament light chain (NFL) levels (in pg/ml) were examined.

Cardiac markers were collected from EFACTS registry records to provide a thorough overview of cardiac involvement in FA patients. These markers encompassed a diverse range of clinical, echocardiographic, and electrocardiographic data. We included clinical data, e.g., the presence of cardiac symptoms (syncope, dyspnea, palpitations, and chest pain), hypertension, cardiac hypertrophy, arrhythmias, ischemic cardiopathy, and other types of cardiopathy, and the age of onset for these symptoms. We added the structural aspects of cardiac function in FA patients by intraventricular and posterior wall thickness, recorded in millimeters, and the ejection fraction, quantified as a percentage. Electrocardiographic data like the identification of sinus rhythm, repolarization abnormalities, Q-waves, arrhythmias, left ventricular hypertrophy, conduction abnormalities, and the pacemaker’s implantation were used.

### Key laboratory methods

Blood samples to assess activities of ETC complexes (NQR, SQR, and COX) and Q10 levels were collected at Motol University Hospital and analyzed at the Department of Pediatrics and Inherited Metabolic Disorders, Charles University, using chemicals from Sigma-Aldrich and a TANAKA Q10 standard. For the assays, 9 ml of peripheral blood was utilized for platelet isolation, and 2 ml of Li/heparin-treated blood for Q10 quantification.

Platelet isolation was achieved through differential centrifugation, adapted from Fox et al. [41].

The enzymatic activities of the respiratory chain complexes and citrate synthase (CS) were spectrophotometrically measured following protocols from Rustin [42] and Srere [43], respectively. The use of CS as a marker for the number of healthy mitochondria [35, 36] is crucial in this context (the COX/CS ratio was counted).

Total Q10 in plasma was determined via Mosca's method [44], which incorporates extraction and high-performance liquid chromatography (HPLC) analysis.

All assessed enzyme activities were normalized against cellular protein content. For protein quantification, we utilized the Lowry method [45].

Neurofilament light chain (NFL) levels were measured with an NF-light™ Serum ELISA RUO kit [46] employing a 3,3´, 5,5´-tetramethylbenzidine (TMB) substrate for colorimetric Enzyme-Linked Immunosorbent Assay (ELISA).

The chemiluminescent immunoassay (CMIA) method [47, 48] was used to quantify serum myoglobin (in µg/L) for early diagnosis of myocardial infarction and muscle-related conditions, and N-terminal Pro-Brain Natriuretic Peptide (NT-proBNP, in ng/L) for monitoring heart dysfunction.

For detailed protocols and comprehensive methodological information, please refer to the online supplement.

### Artificial intelligence generated content (AIGC) tool

The language of this manuscript was enhanced using Chat Generative Pre-Trained Transformer (ChatGPT) version 4, an AIGC tool, which assisted in refining grammar, and improving phrasing.

### Statistical analyses

Statistical procedures were tailored to the study's observational design, focusing on comparisons between FA patients and controls, and exploring associations with clinical variables. Our statistical analyses were conducted using R version 4.3.1. The foundational design of our approach involved comparing FA patients with healthy controls across all pre-defined clinical and laboratory parameters. In addition, we examined intricate correlations within these parameters, such as disease severity and cardiovascular markers.

For initial comparisons, we primarily employed two-tailed Welch's t-tests to assess the means of continuous variables between the FA patient group and the healthy control group. Benjamini–Hochberg method was applied for multiple testing correction. These comparisons are detailed in Table 1. To further explore the data, we utilized linear regression models with a twofold purpose. First, these models were applied to assess the interrelations among mitochondrial markers themselves, such as NQR, COX, SQR, CS, and Q10 levels. Second, we explored associations between these ETC enzyme activities and a variety of clinical parameters, including demographics, clinical markers, and cardiovascular symptoms. We applied the chi-square test of independence to evaluate the association between gender and health status within our study groups.

**Table 1:** Compares demographic and clinical characteristics between FA patients (P) and healthy controls (HC), encompassing parameters like gender, age at examination, and specific metrics for FA patients such as disease onset, duration, GAA repeats, and various severity indices. Detailed statistical data including means, standard deviations, ranges, and participant counts are presented. The Chi-square test showed no significant gender-based differences in health status (p=0.8423), and age comparisons revealed similar distributions across both cohorts

Parameter	P mean ± SD (range min–max) [*N*]	HC mean ± SD (range min–max) [*N*]
Gender (male:female) *p* = 0.84	19:15	9:8
Age at Examination (years) *p* = 0.90	31 ± 12 (7–55) [*N* = 34]	30 ± 13 (13–59) [*N* = 17]
Age at Onset (years)	12 ± 6 (1–33) [*N* = 34]	NA
Disease Duration (years)	20 ± 10 (4–40) [*N* = 34]	NA
GAA1 Repeats	647 ± 266.51 (90–1160) [*N* = 34]	NA
GAA2 Repeats	884 ± 263.65 (150–1330) [*N* = 33]	NA
Point Mutation	1 [*N* = 1]	NA
Disability Stage*	5 ± 1.51 (2–7) [*N* = 34]	NA
Severity Index	0.36 ± 0.23 (0.15–1.00) [*N* = 31]	NA
Age of Permanent Wheelchair Use (years)	23 ± 8.77 (9–46) [*N* = 23]	NA
Age of Permanent Walking Aid Use (years)	21 ± 9.94 (5–46) [*N* = 26]	NA
ADL Sum	16 ± 7.29 (2–27) [*N* = 34]	NA
SARA Sum	23 ± 10.57 (7–40) [*N* = 34]	NA
MoCA Total	26 ± 4.08 (9–30) [*N* = 28]	NA
Avg. Annual Worsening in ADL	0.96 ± 0.47 (0.30–2.42) [*N* = 34]	NA
Avg. Annual Worsening in SARA	1.39 ± 0.68 (0.47–3.29) [*N* = 34]	NA

^*^scale 1–7, where 1 means “no functional handicap but signs at examination” and 7 means “confined to bed”

Each linear regression model provided detailed outputs, including regression coefficient estimates, a 95% confidence interval (CI), and corresponding p-values, adopting a significance level threshold of 0.05. Additionally, we reported the R^2^ (r) value and the total count of observations (N) for each model. Our models incorporated indicators for patient and control status and their interaction with the explanatory variables. However, in specific models where data were available only for the patients, these variables were omitted. Individual p values were adjusted for multiple testing using Benjamini–Hochberg method. Multiple testing corrections were done for each aim separately.

This dual approach in our statistical methodology enabled us not only to compare FA patients with controls but also to uncover potentially significant correlations related to disease burden, and cardiac markers, employing the same robust statistical methods for all comparisons.

## Results

The study involved a detailed examination of mitochondrial functions in 34 FA patients, referenced as 'P' in tables and charts, and 17 healthy controls, referenced as 'HC.' Table X1A and Table X1B in online supplement provide detailed descriptions of both, the healthy control and FA patient cohorts, respectively.

There were no significant differences in age and gender ratio between the healthy control group (HC) and the FA patient group (P), with p values exceeding 0.84 and 0.90 respectively, as shown in Table 1. The primary clinical characteristics of the FA patient cohort, including disease onset age, disease duration, GAA1 and GAA2 repeats expansions, point mutations, disability stage, severity index, age at permanent wheelchair use, age at permanent walking aid use, and scales measuring disease progression (such as ADL sum, SARA sum, annual average ADL gain, annual average SARA gain, and MoCA total), are also detailed in Table 1.

In Table 2, we compared data from the laboratory assessment of NQR, SQR, COX, CS, COX/CS ratio, and plasma Q10 levels. Additionally, we also analyzed NFL levels, along with selected cardiomarkers (myoglobin and NT-proBNP).

**Table 2:** Each parameter for FA patients (P) and healthy controls (HC) is presented along with its mean value, standard deviation, range (minimum-maximum), and the number of subjects evaluated [*N*]. Additionally, the *p*-values are provided to indicate the statistical significance of differences observed between the two groups. In bold are statistically significant results

Analytical Results	*P* mean ± SD (range min–max) [N]	HC mean ± SD (range min–max) [N]	*p*-value	*p*-value adjusted
NQR (nmol/min*mg prot)	46.47 ± 15.07 (19–75.2) [*N* = 26]	61.83 ± 30.15 (21.7–132) [*N* = 17]	0.065	0.092
SQR (nmol/min*mg prot)	12.55 ± 3.54 (6–22.26) [*N* = 28]	20.70 ± 7.75 (8.5–37.97) [*N* = 16]	0.001	**0.002**
Q10 (µg/ml)	0.86 ± 0.39 (0.33–2.28) [*N* = 34]	0.65 ± 0.26 (0.36–1.21) [*N* = 16]	0.032	0.064
Q10^ (µg/ml)	0.78 ± 0.29 (0.33–1.49) [*N* = 29]	0.65 ± 0.26 (0.36–1.21) [*N* = 16]	0.128	0.160
COX (nmol/min*mg prot)	12.68 ± 5.07 (6.38–26.48) [*N* = 28]	22.36 ± 5.95 (9.75–31.03) [*N* = 17]	< 0.001	** < 0.001**
CS (nmol/min*mg prot)	72.27 ± 14.09 (35.75–103.62) [*N* = 28]	73.23 ± 7.69 (59.08–90.99) [*N* = 17]	0.771	0.771
COX/CS ratio	0.18 ± 0.07 (0.09–0.37) [*N* = 28]	0.31 ± 0.08 (0.13–0.42) [*N* = 17]	< 0.001	** < 0.001**
NFL (pg/ml)	17.65 ± 10.39 (0.74–50.91) [*N* = 33]	6.11 ± 2.42 (1.9–9.84) [*N* = 12]	< 0.001	** < 0.001**
Myoglobin (µg/l)	39.74 ± 16.57 (13.5–84.7) [*N* = 34]	42.34 ± 14.83 (25.3–79.3) [*N* = 13]	0.607	0.675
NT-proBNP (ng/l)	107.35 ± 156.35 (26.4–813) [*N* = 34]	51.52 ± 28.07 (35–132.7) [*N* = 13]	0.053	0.088

^ Excluded the 5 FA patients who reported Q10 supplementation

Activities of mitochondrial NQR, SQR, and COX, Q10 levels, and CS activity are outlined in Table X2A and X2B of the online supplement, which provide a comprehensive overview of enzymatic activities and biochemical levels for the healthy control and FA patient cohorts. The tables specifically address activity measures for NQR, SQR, COX, and CS in nmol/min*mg protein. Additionally, the COX/CS ratio is calculated and plasma Q10 levels in µg/ml are included. Statistical attributes, such as the number of observations (N), mean, highest value (max), and lowest value (min), for each assessed parameter are also presented.

**Fig. 2:**
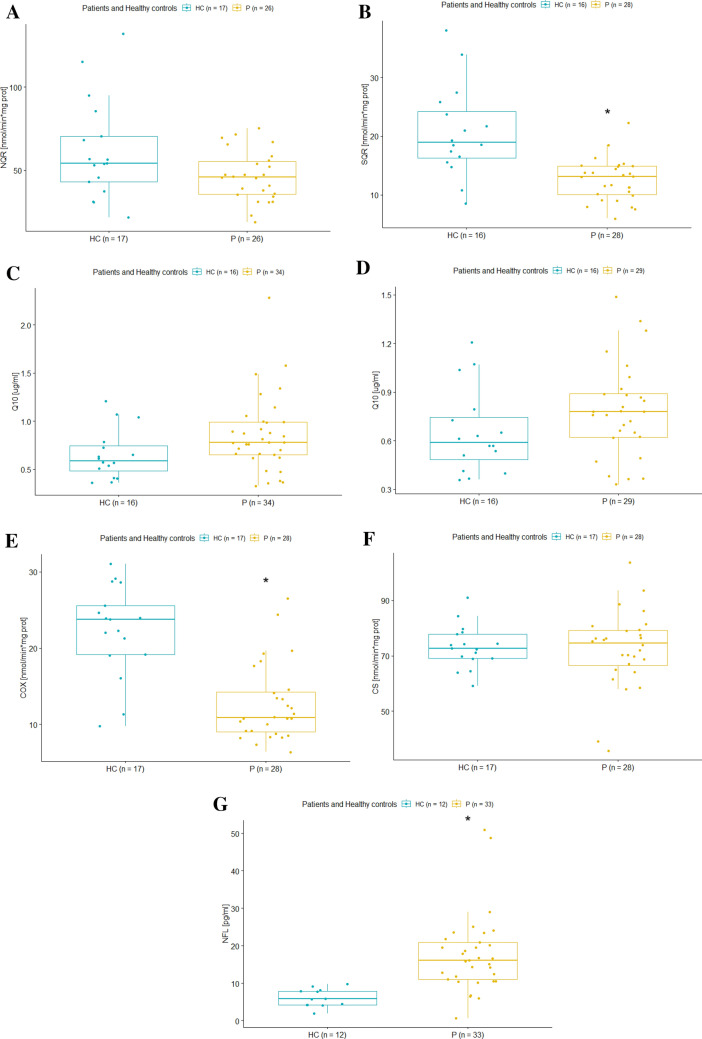
Chart matrix 1A-1G: Comparative Box Plots of Key Electron Transport Chain Enzymatic Activities and Biomarkers in FA Patients and Healthy Controls. Charts 1A-1G present a series of box plots that visually represent the distributional characteristics of six key variables in the study of FA and a Healthy Control (HC) group. These variables include NQR activity (Chart 1A), SQR activity (Chart 1B), Coenzyme Q10 (Q10) levels (Chart 1C), Adjusted Q10 levels, excluding data from five FA patients (P) on Q10 supplementation (Chart 1D), COX activity (Chart 1E), CS activity (Chart 1F), and Neurofilament light chain (NFL) levels (Chart 1G). Each chart is structured to compare the FA patient cohort (P) with the Healthy Control group (HC), facilitating a clear visual comparison between the two groups. The horizontal axis of each plot labels the cohorts, while the vertical axis represents the measured values of enzyme activities or biomarker levels. The ensemble of box plots allows for an immediate visual grasp of differences or similarities in these parameters between the FA and HC groups. Complementing these visual representations, Table 2 provides detailed statistical data, including p-values, to support the observed distributional trends

As shown in Table 2 and in Fig. 2 (Chart matrix 1A-1G), there was a trend toward reduced NQR activity in FA patients compared to healthy controls, though it did not reach conventional statistical significance (*p* = 0.092). A significant decrease in SQR activity was observed in FA patients (*p* = 0.002). The COX activity shows highly significant reductions in FA patients (*p* < 0.001). CS activity did not vary significantly between FA patients and healthy controls (*p* = 0.771). The COX/CS ratios is highly significantly reduced (*p* < 0.001). We show tendency to elevated Q10 levels in FA patients, the difference is statistically insignificant (*p* = 0.064), even more when excluding 5 FA patients with reported Q10 supplementation (*p* = 0.160).

We offer an extension to our main study by featuring longitudinal data on mitochondrial functions for three patients from the FA patient cohort, assessed approximately 17–19 years prior, which are showed in the Table 3, in which we present comparisons of these three FA patients’ characteristics and available enzymatic activities measured at two different time points. The enzymatic activities in COX declined during the disease progression, trends in other enzyme activities differ between the three patients.

**Table 3:** Longitudinal data in 3 FA patients (P) 17–19 years apart reveal a general trend of decreasing activity in Complex IV (COX) enzyme across all three patients. On the other hand, two of the three patients exhibited an increase in Complex II (SQR) activity. Given these intriguing shifts in enzymatic activity over time, further investigation is certainly warranted to interpret these results fully

FA patient yob	1978				1990				1985			
Age at Examination	44				31				36			
Gender	1				2				2			
GAA1	200				1100				500			
GAA2	300				1160				1160			
Point Mutation	none				none				none			
Visit	**0**	**1**			**0**	**1**			**0**	**1**		
Blood Draw	**ID 109**	**ID 37**	*delta*	*%*	**ID 117**	**ID 5**	*delta*	*%*	**ID 118**	**ID 18**	*delta*	*%*
Blood Draw Age	**25**	**42**	*17*		**12**	**31**	*19*		**18**	**37**	*18*	
NQR	NA	39.20	*NA*	*NA*	34.80	31.04	***− ****3.8*	*89%*	45.30	47.31	*2.0*	*104%*
SQR	NA	9.10	*NA*	*NA*	8.20	14.86	*6.7*	*181%*	9.50	11.52	*2.0*	*121%*
COX	**26.20**	**18.29**	***− 7.9***	***70%***	**24.20**	**13.44**	***− 10.8***	***56%***	**22.60**	**19.30**	***− 3.3***	***85%***
CS	88.40	61.54	***− ****26.9*	*70%*	64.40	67.05	*2.6*	*104%*	69.00	64.97	***− ****4.0*	*94%*
COX/CS	0.30	0.30	*0.0*	*100%*	0.38	0.20	***− ****0.2*	*53%*	0.33	0.30	*0.0*	*92%*

Gender: 1 = male, 2 = female

In our linear regression analysis on assessed ETC and TCA enzymes (NQR, SQR, COX, CS) and Q10 levels, only a few significant effects were noted between them. We observed a significant (*p* < 0.001) link between COX activity and the COX/CS ratio, as seen in Table 4.

**Table 4:** Presents a detailed comparison of citrate synthase (CS), complexes I (NQR), II (SQR), and IV (COX), COX/CS ratio, alongside coenzyme Q10 (Q10) levels, between FA patients (P) and healthy controls (HC). The data derived from linear regression models showcased the relationships and interactions among these variables, we present significant and interesting results. Notably, the table introduces an interaction term, 'Is_patient', which examines the influence of patient status on the relationship between selected parametres

Endogenous variable	*R*^2^ (r)	*N*	Regressor	Coefficient	95% Confidence Interval (CI)	*p*-value	*p*-value adjusted
**COX**	0.867	45	intercept	0.717	(− 4.917, 6.351)	0.799	0.799
			**COX/CS**	70.918	(52.975, 88.860)	** < 0.001**	** < 0.001**
			Is_patient	1.341	(− 4.951, 7.634)	0.669	0.765
			**COX/CS: Is_patient**	− 12.261	(− 35.259, 10.737)	0.228	0.461
**CS**	0.193	44	intercept	68.063	(51.328, 84.798)	** < 0.001**	** < 0.001**
			**SQR**	0.251	(− 0.509, 1.011)	0.508	0.677
			Is_patient	− 18.963	(− 42.222, 4.295)	0.107	0.214
			**SQR: Is_patient**	1.595	(0.140, 3.049)	**0.032**	0.086

We also describe differences in relationships between SQR and CS in FA patients in comparison to healthy controls. The 'SQR: Is_patient' interaction term in our analysis indicates a borderline insignificant (*p* = 0.086) difference in the effect of SQR on CS activity when comparing FA patients and healthy controls. The positive coefficient (1.595) suggests that in FA patients, a change in SQR activity is associated with a roughly 7–8 times greater change in CS activity compared to controls. The charts in an online supplement in Figure X1 (Chart matrix X1A-X1C) demonstrate this relationship.

Our comprehensive linear regression analysis assessing NQR, SQR, COX, and CS relationships with clinical, laboratory and disease severity metrics surprisingly revealed only non-significant results. Q10 was excluded from the next linear regression analyses with clinical parameters due to the patient uncertainty regarding their Q10 supplementation status. This decision was not part of the original study plan but was necessary to ensure the accuracy and reliability of our findings.

We conducted an in-depth analysis centered on cardiac markers, utilizing patient history (encompassing the onset of symptoms), laboratory tests, and cardiological evaluations. Table 2 includes the comparative data of cardiac and muscle laboratory biomarkers between FA patients and healthy controls. The average levels of NT-proBNP, a marker of atrial dilation in heart failure, show some tendency to be elevated in FA patients compared to healthy controls (p = 0.088), but only rarely to pathological levels (above 125 ng/l), as is visible in the supplemental Table X3A and X3B in online resources. Myoglobin levels in FA patients tend to be lower than in healthy controls (p = 0.04) as is also presented in the Table 2 and Tables X3A and X3B online.

**Fig. 3:**
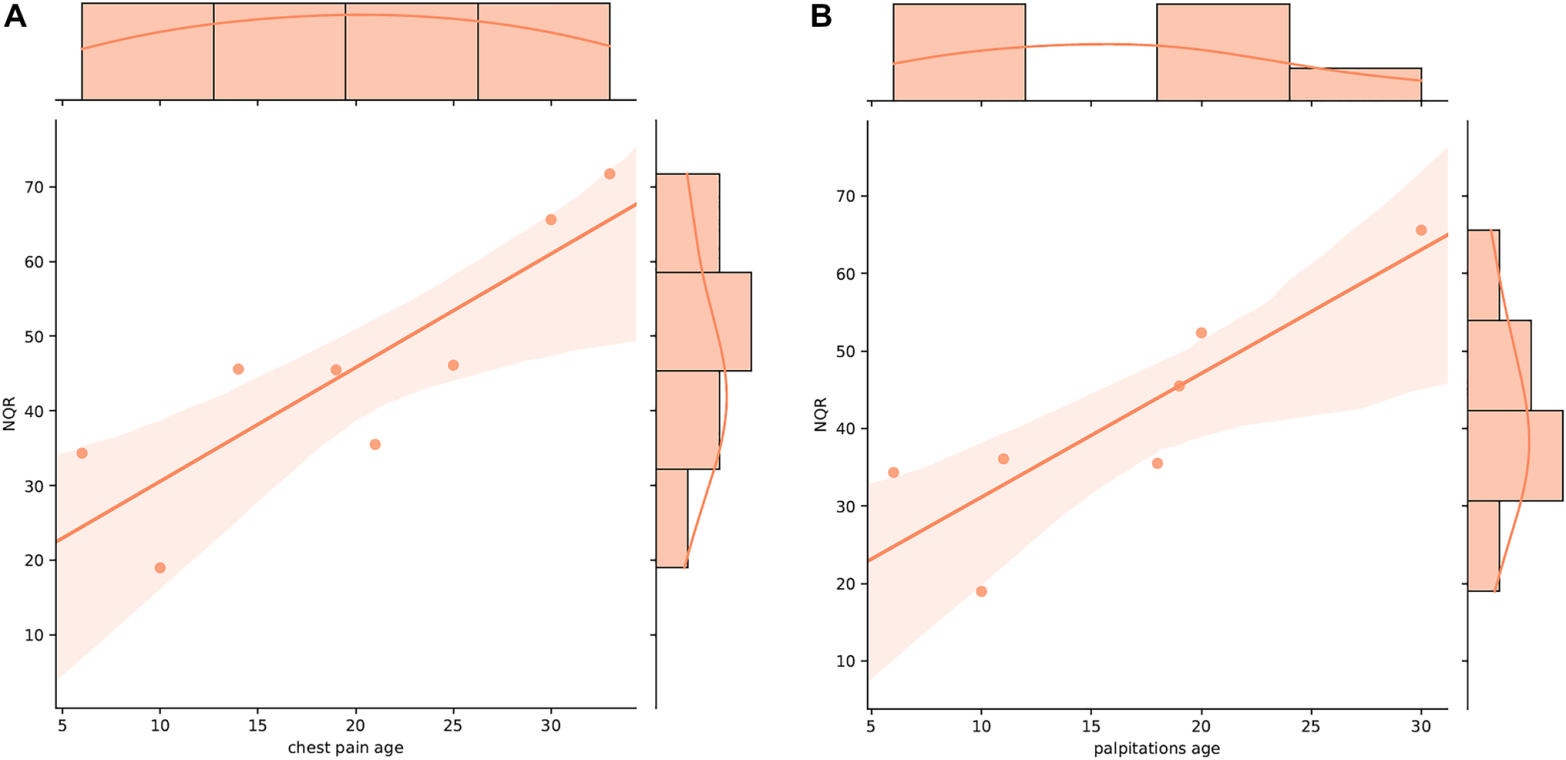
Chart Matrix 2A-2B: Influence of Cardiological Symptom Onset on NADH:Quinone Oxidoreductase Activity. In Charts 2A and 2B, we present the association between the age at onset of cardiological symptoms and Complex I (NQR) activity, a pivotal enzyme in the mitochondrial electron transport chain, within our FA patient (P) cohort. The analysis reveals a discernible pattern where an earlier onset of chest pain and palpitations in years correlates with reduced NQR activity in nmol/min*mg protein (chest pain: *r* = 0.7209, *p* < 0.008; palpitations: *r* = 0.7344, *p* < 0.014). This suggests that mitochondrial dysfunction, as indicated by NQR activity in platelets, may be more pronounced in patients who experience cardiac symptoms at an earlier stage of the disease. While these findings are statistically significant, the interpretations are made cautiously given the limited sample size, emphasizing the need for further studies to confirm these observations and understand their implications for the pathophysiology of FA

In Table X3B in online resources, we show that among the 28 FA patients, 8 reported experiencing chest pain and 7 cited palpitations. Figure 3 (Charts matrix 2A and 2B) and Table 5 demonstrate that onset age of these symptoms notably influenced NQR activity for the chest pain (*r* = 0.7209, *p* < 0.05) and the palpitations (*r* = 0.7344, *p* < 0.05) showing significant effects. The sooner FA patients reported above-mentioned cardiac symptoms, and the lower activity of the NQR was observed. Myoglobin blood levels showed a relationship with NQR, which is statistically significant after adjusting (*p* = 0.018).

**Table 5:** Presents the results of a linear regression analysis examining the influence of cardiological variables on the activities of studied TCA’s and ETC’s enzymes in FA patients

Endogenous variable	*R*^2^ (r)	N	Regressor	Coefficient	95% Confidence Interval (CI)	*p*-value	*p*-value adjusted
**COX**	0.542	8	intercept	17.771	(11.506, 24.036)	** < 0.001**	**0.002**
			**palpitations_age**	− 0.366	(− 0.703, − 0.030)	**0.037**	0.068
**CS**	0.216	41	intercept	79.503	(59.493, 99.512)	** < 0.001**	** < 0.001**
			**Myoglobin**	− 0.129	(− 0.577, 0.319)	0.563	0.563
			Is_patient	− 23.738	(− 46.853, − 0.624)	**0.044**	0.071
			**Myoglobin:Is_patient**	0.560	(0.032, 1.088)	**0.038**	0.068
**NQR**	0.734	7	intercept	15.214	(− 4.529, 34.957)	0.105	0.139
			**palpitations_age**	1.596	(0.493, 2.699)	**0.014**	**0.037**
**NQR**	0.721	8	intercept	15.352	(− 5.135, 35.840)	0.116	0.143
			**chest_pain_age**	1.523	(0.576, 2.469)	**0.008**	**0.025**
**NQR**	0.356	39	Intercept	16..021	(− 19.153, 51.195)	0.362	0.413
			**Myoglobin**	1.181	(0.394, 1.968)	**0.004**	**0.018**
			Is_patient	16.925	(− 24.106, 57.956)	0.408	0.435
			**Myoglobin:Is_patient**	− 0.819	(− 1.765, 0.128)	0.088	0.128

This comprehensive analysis incorporates endogenous variables, such as citrate synthase (CS), complexes I (NQR), II (SQR), and IV (COX), alongside key clinical factors (regressors). The table highlights only the statistically significant findings, which have been adjusted for multiple testing corrections, emphasizing notable relationships like the onset age of palpitations and myoglobin levels. The 'Is_patient' regressor, indicating whether the subject is an FA patient, is used to differentiate the impact on enzyme activities between FA patients and healthy controls to highlight any unique enzyme activity patterns in FA patients, offering insights into the disease-specific metabolic alterations

Our analysis showed in Table 2, a highly significant (*p* < 0.001) difference in serum NFL concentrations between FA patients and controls, with higher mean levels in the patient group. In an online supplement in Table X4, the regression analysis reveals that with increasing age, there is a statistically significant decrease in NFL levels, suggesting that age may be inversely correlated with the severity of neurodegeneration in FA patients, as older individuals showed approximately 0.4 pg/ml lower NFL levels per year of age.

Our study's thorough examination of the relationships between clinical parameters and mitochondrial markers, despite being conducted in a moderately sized cohort, incorporates multiple comparison corrections. These adjustments have led to the exclusion of certain positive outcomes, reducing Type I errors (false positives).

## Discussion

To our knowledge, this cross-sectional observational study represents one of the most extensive analyses of mitochondrial enzyme activities in blood samples from FA patients prior to the initiation of incoming targeted therapy. In this study, we evaluated plasma Q10 levels in 34 FA patients and explored mitochondrial TCA (CS) and ETC (NQR, SQR, COX) enzyme activities in 28 of these individuals, selected from 41 Czech patients registered in the EFACTS registry. FA patient cohort included homozygotes with both GAA repeat expansions above 80, and one heterozygous FA patient with both a point mutation and GAA repeat expansion. Our study also assessed biosamples from 17 demographically nearly matched healthy controls. The study revealed disparities in mitochondrial enzyme activities between FA patients and healthy individuals.

Notably, SQR and COX activities were markedly reduced in FA patients, showing significant differences with p values of 0.002 and < 0.001, respectively. NQR, on the other hand, displayed a trend toward reduction, though it did not reach statistical significance (*p* = 0.092). Interestingly, NQR requires a higher number of Fe–S clusters (7–8), compared to SQR (3), as illustrated in Fig. 1. This observation contrasts with recent findings that emphasize a significant impact of frataxin deficiency on NQR in FA cells [21], whereas our study observed a more pronounced reduction in SQR and especially in COX activity, which was not evaluated by the mentioned study [21]. Moreover, the substantial decrease in COX activity, an enzyme not directly dependent on Fe–S clusters, indicates additional underlying pathophysiological mechanisms in FA.

By comparing the activity of COX to CS, which serves as a marker for the number of healthy mitochondria, we aimed to discern between potential suppression of ETC complex activity and alterations in the number of mitochondria, possibly due to failed mitochondrial biogenesis or genetic regulation. Our observation indicates that COX activity is a significant determinant of the COX/CS ratio, especially when CS activity in platelets is consistent between FA patients and healthy individuals, highlighting the nuanced role of COX in mitochondrial function within the context of FA.

A notable reduction in COX activity could be attributed to increased ROS production or other detrimental molecules, reduced NQR and SQR activities, or alterations in metabolic pathways and epigenetic factors in FA patients. Considering a published study [49], which reported heightened lipid peroxidation in FA model neuronal cells, this phenomenon could elucidate the reduced COX activity observed. The study found that lipid peroxidation could lead to up to a 50% decrease in COX activity, which aligns with our observations. Another study [50] supports this result, demonstrating that mitochondrial energy imbalance and lipid peroxidation precipitate in cellular death in FA. Therefore, these findings collectively emphasize the importance of lipid peroxidation as a pathological mechanism in FA, potentially leading to decreased COX activity and subsequent cellular energy deficits. Additional contributory factors must be considered, such as oxidative stress or interconnected OXPHOS pathway defects, as supported by literature [51, 52]. The formation of respiratory supercomplexes, which involves complexes I, III, and IV, may further influence these dynamics [53, 54]. In addition to the above-mentioned, COX activity impairment in FA can be attributed to disrupted heme biosynthesis, linked to Fe–S cluster protein deficiency [55].

Recent study [56] highlighted significant proteomic changes in FA, suggesting an intricate network of biochemical events linked to frataxin deficiency. Similarly, other study [57] provided insights into the altered transcriptomic and proteomic profiles in FA, particularly in proprioceptive neurons, illustrating the widespread impact of frataxin loss. Furthermore, [58] observed distinct proteomic alterations in the skeletal muscle of FA patients, indicating a broad spectrum of mitochondrial dysfunction, clearly also the Nrf2 pathway disruption, which is a target of newly approved FA treatment (omaveloxolone). Our study findings of decrease in the activities of COX and SQR in platelets from FA patients align with these findings. These findings indicate different adaptive cellular responses and a more complex FA pathology extending beyond frataxin's known functions and Fe–S cluster formation deficiency.

Regarding the Q10 levels, we revealed a non-significant elevation among FA patients compared to controls (*p* = 0.064), with the difference becoming even less significant when considering Q10 supplementation (*p* = 0.160). The study's findings underscore the need to consider Q10 supplementation history in FA, as it notably influences gene expression related to COX subunits and stress response pathways, according to a mouse study [59]. Although Q10 levels are elevated, potentially as a compensatory mechanism, this increase does not appear to significantly affect the activities of other ETC complexes.

Although the literature hinted at a possible change in CS activity in FA patients’ calf muscles [19] and skin fibroblasts, [34], our observations in platelets (Table 2) found no significant deviations in CS activity (*p* = 0.771) from the blood samples between FA patients and healthy controls. Our analysis identified a trend suggesting a differential effect of SQR on CS activity between FA patients and healthy controls. However, this difference became statistically non-significant after adjustment (*p* = 0.086). While not conclusive, this trend remains noteworthy, especially considering SQR's role in the TCA cycle. SQR's involvement in transferring electrons to Q10 during the conversion of succinate to fumarate, a process influenced by frataxin through succinate dehydrogenase (SDH), as indicated by [60], provides crucial insights. A change in SQR activity is associated with a roughly 7–8 times greater change in CS activity compared to controls. This suggests a more pronounced effect of SQR activity on CS in FA patients, particularly in the interplay between the ETC and TCA cycle, as evidenced by previous studies [11, 12, 22, 55–58, 60].

In terms of correlating the activity of ETC enzymes with various clinical metrics including GAA expansions, our efforts were largely inconclusive. Only subtle effects were discerned, and these findings could potentially be influenced by the limited sample size.

In a subset (*N *= 7–9) of FA patients (Fig. 3), we observed a robust positive correlation between reduced NQR activity and the age of onset of palpitations and dyspnea, which are common clinical symptoms of cardiac pathology. These findings highlight a potentially significant role of NQR and a more subdued contribution of COX to these cardiac manifestations in FA patients. While the direct relationship between OXPHOS system dysfunction and FA-induced cardiomyopathy is yet to be comprehensively studied, existing literature has pointed toward an association between disruptions in OXPHOS enzymes and various forms of cardiomyopathies incl. FA cardiomyopathy, as was previously mentioned in the introduction part [24–27]. This opens promising avenues for future research to explore and understand these connections more deeply.

The study noted the tendency toward elevated levels of NT-proBNP, a recognized indicator of atrial dilation in cardiac failure, in FA patients, approaching statistical significance (*p* = 0.088). Pathological levels indicative of heart failure with a diminished ejection fraction below 40% typically exceed 357 pmol/l [61]. This is roughly equivalent to 3000 ng/l when considering the molecular weight of NT-proBNP at 8.5 kDa. For reference, the standard range for NT-proBNP is for most of the patients based on their age between 35.0 and 125.0 ng/l. In our patient group, six FA patients exhibited levels above this normal range, with the highest concentration reaching 813 ng/l (in an individual exhibiting recurrent ventricular tachycardia). In stark contrast, the established cardiac markers, and echocardiographic metrics, such as IVS, PWd, and EF, which are commonly used for FA management [6], showed no significant association with the enzymatic activities listed.

Slightly lower myoglobin levels in FA patients compared to healthy controls, indicative of muscle atrophy as previously established [62], suggest that mitochondrial dysfunction in FA leads to disruptions in energy production, ultimately resulting in muscle damage. This can manifest both in the form of heightened myoglobin concentrations during abrupt cell death events or reduced levels during chronic muscle atrophy, a scenario more likely in FA. It's essential to note a limitation of this finding: our analyses were limited to platelets for assessing TCA and ETC complexes activities and serum/plasma levels of myoglobin. This focus may introduce a bias, considering platelets' unique mitochondrial content might not fully reflect the cellular and molecular dynamics of muscle tissues. The recent research on frataxin isoforms, particularly the differences observed between erythrocytes and other tissues [63, 64], underscores the importance of considering tissue-specific disparities in mitochondrial function. This variance is crucial when interpreting our results, as it highlights the complexity of mitochondrial pathology in different cellular environments. Therefore, a careful and nuanced interpretation of our findings is necessary.

We observed a significant elevation in serum NFL levels in FA patients (*p* < 0.001), aligning with previous research findings [37–40]. However, this elevation did not correlate with the activities of ETC complexes, suggesting that while NFL levels may indicate disease presence, they do not directly reflect mitochondrial dysfunctions associated with FA. The negative correlation between NFL levels and patient age, detailed in Table X4 of the online supplement, indicates lower NFL levels in older patients and higher levels in younger ones. This suggests NFL's role in reflecting FA's progression. Possible explanations include more active axon remodeling in younger patients, axonal loss over time leading to decreased serum NFL levels, or earlier demise of patients with advanced disease and higher NFL levels [37–40]. This finding prompts further investigation into the role and implications of NFL in FA beyond the scope of mitochondrial activity and this study.

Our study presents a comprehensive analysis of mitochondrial enzyme activities in a cohort of FA patients, with an average disease duration of 20 years, primarily consisting of older individuals. Despite the older average age of our cohort, our study also encompassed a diverse range of disease durations, including ambulatory patients and those with less than ten years of progression. This diversity allowed for a balanced analysis across various stages of FA.

Importantly, we found no significant association between disease duration and mitochondrial enzyme levels in our analyses, with only a minor decrease in COX activity noted in a subset of three patients over nearly two decades. This observation is in line with our previous research [65], which showed no significant age-related differences in electron transport chain and citrate synthase activities in platelets across different ages. Given the rapid turnover of platelets, our measurements reflect the current mitochondrial status at the time of collection.

Our research offers a comprehensive analysis within its established scope; nevertheless, it encounters specific methodological and contextual limitations. The observational cross-sectional design of the study inherently constrains our ability to establish causality. Furthermore, the relatively modest size of our Czech FA cohort limits the generalizability of our findings. Notably, we observed correlations between a limited set of mitochondrial function parameters and clinically relevant markers. These observations were primarily confined to analyses conducted on platelets for assessing TCA and ETC complexes activities. Additionally, the reliance on serum/plasma levels for other compounds introduces further limitations, as different tissues might yield divergent results. These constraints, especially the limited cohort size and the tissue-specific nature of our analysis, impede the immediate application of our findings in clinical trial settings. Therefore, there is a compelling need for validation through larger, multicentric studies, which would enhance the external validity and clinical applicability of our results.

In summary, this study stands as one of the most extensive analyses of mitochondrial enzyme activities in FA patients, contributing significantly to our understanding of FA's pathophysiology. It also paves the way for future research, particularly in validating the efficacy of enzymes like COX, SQR, and NFL as clinical and treatment indicators in FA. The observed differences between patients and healthy controls highlight the potential for mitochondrial function improvement during therapy, warranting further investigation to confirm their role in FA clinical management.

### Supplementary Information

Below is the link to the electronic supplementary material.

Supplementary file1 (DOCX 172 KB)

## Data Availability

The data presented in this study are available as part of the online supplementary materials - Tables starting with X mark. Further inquiries regarding the data can be addressed to the corresponding author. Please note that the data are derived from a small cohort of 28 patients, raising up the ethical concerns to publish the extended dataset.

## Abbreviations

ADL: Activities of Daily Living
ATP: Adenosine Triphosphate
AVG ADL: Average Activities of Daily Living per year of disease duration
AVG SARA: Average Scale for the Assessment and Rating of Ataxia per year of disease duration
COX: Cytochrome c Oxidase (Complex IV)
CS: Citrate Synthase
EF: Heart Ejection Fraction
EFACTS: European FA Consortium for Translational Studies
ELISA: Enzyme-Linked Immunosorbent Assay
EMA: European Medicines Agency
ETC: Electron Transport Chain
FA: Friedreich's Ataxia
FDA: Food and Drug Administration
Fe–S: Iron–Sulfur (clusters)
FXN: Frataxin; GAA1/GAA2—shorter/longer allele with Guanine-Adenine-Adenine repeats
HC: Healthy Control
ID: Identifier
IVS: Interventricular Septum Thickness
MoCA: Montreal Cognitive Assessment
N: Number of probands
NA: Not available
NADH: Nicotinamide Adenine Dinucleotide (reduced form)
NFL: Neurofilament Light Chain
NQR: NADH:Quinone Oxidoreductase (Complex I)
Nrf2: Nuclear factor erythroid 2-related factor 2
NT-proBNP: N-Terminal pro b-type Natriuretic Peptide
OXPHOS: Oxidative Phosphorylation
P_HC: FA Patient (P) vs. Health Control (HC) identifier
pNFH: Phosphorylated Neurofilament Heavy Chain
PWd: Posterior Wall Thickness (diastolic)
Q10: Coenzyme Q10, also known as Ubiquinone (Q from Quinone)
QCCR: Ubiquinol-Cytochrome c Oxidoreductase (Complex III)
ROS: Reactive Oxygen Species
SARA: Scale for the Assessment and Rating of Ataxia
SD: Standard Deviation
SQR: Succinate:Quinone Oxidoreductase (Complex II)
TCA cycle: Tricarboxylic Acid Cycle = Krebs Cycle
yob: Year of Birth
