# Supplementary material for: Exploring mitochondrial biomarkers for Friedreich's ataxia: a multifaceted approach
Source: J Neurol. 2024 Mar 23;271(6):3439–54. doi: 10.1007/s00415-024-12223-5 (PMC11136723; doi:10.1007/s00415-024-12223-5)
Supplement: Supplementary file 1 — Supplementary file1 (DOCX 172 KB) [file 415_2024_12223_MOESM1_ESM.docx]

Online Resources – Supplement

Title Exploring Mitochondrial Biomarkers for Friedreich's Ataxia: A Multifaceted Approach

Authors Stovickova Lucie^1,7^, Hansikova Hana^2^, Hanzalova Jitka^3^, Musova Zuzana^4,7^, Semjonov Valerij^8^, Stovicek Pavel^9^, Hadzic Haris^5^, Novotna Ludmila^5^, Simcik Martin^5^, Strnad Pavel^5^, Serbina Anastaziia^5^, Karamazovova Simona^6,7^, Schwabova Paulasova Jaroslava^6,7^, Vyhnalek Martin^6,7^, Krsek Pavel^1^, Zumrova Alena^1,7^

Affiliations

^1^ Department of Paediatric Neurology, Second Faculty of Medicine, Charles University, Motol University Hospital, Prague 5, Czech Republic;

^2^ Department of Paediatrics and Inherited Metabolic Disorders, First Medical Faculty, Charles University and General University Hospital in Prague, Prague 2, Czech Republic;

^3^ Department of Immunology, Second Faculty of Medicine, Charles University, Motol University Hospital, Prague 5, Czech Republic;

^4^ Department of Biology and Medical Genetics, Second Faculty of Medicine, Charles University, Motol University Hospital, Prague 5, Czech Republic;

^5^ Second Faculty of Medicine, Charles University, Prague 5, Czech Republic;

^6^ Department of Neurology, Second Faculty of Medicine, Charles University, Motol University Hospital, Prague 5, Czech Republic;

^7^ Centre of Hereditary Ataxias, Second Faculty of Medicine, Charles University, Motol University Hospital, Prague 5, Czech Republic, an official EFACTS site, a member of European Reference Network for Rare Neurological Diseases (ERN-RND);

^8^ Department of Paediatrics, Second Faculty of Medicine, Charles University and Motol University Hospital, Prague, Czech Republic

^9^ Prague, Czech Republic

Authors’ contact details: **Corresponding author**: Stovickova Lucie, MD, Second Faculty of Medicine, Charles University, Motol University Hospital, Centre of Hereditary Ataxias, V Uvalu 84, 15006 Prague 5, Prague, Czech Republic, [lucie.stovickova@fnmotol.cz](mailto:lucie.stovickova@fnmotol.cz), +420 607 081 479, <https://orcid.org/0000-0003-0646-0262>.

Abbreviations

ADL – Activities of Daily Living; ATP – Adenosine Triphosphate; AVG ADL – Average Activities of Daily Living per year of disease duration; AVG SARA – Average Scale for the Assessment and Rating of Ataxia per year of disease duration; COX – Cytochrome c Oxidase (Complex IV); CS – Citrate Synthase; EF – Heart Ejection Fraction; EFACTS – European FA Consortium for Translational Studies; ELISA – Enzyme-Linked Immunosorbent Assay; EMA – European Medicines Agency; ETC – Electron Transport Chain; FA – Friedreich's Ataxia; FDA – Food and Drug Administration; Fe-S – Iron-Sulfur (clusters); FXN – frataxin; GAA1/GAA2 – shorter/longer allele with Guanine-Adenine-Adenine repeats; HC – Healthy Control; ID – Identifier; IVS – Interventricular Septum Thickness; MoCA – Montreal Cognitive Assessment; N – number of probands; NA – Not available; NADH – Nicotinamide Adenine Dinucleotide (reduced form); NFL – Neurofilament Light Chain; NQR – NADH:Quinone Oxidoreductase (Complex I); Nrf2 – Nuclear factor erythroid 2-related factor 2; NT-proBNP – N-Terminal pro b-type Natriuretic Peptide; OXPHOS – Oxidative Phosphorylation; P_HC – FA Patient (P) vs. Health Control (HC) identifier; pNFH – Phosphorylated Neurofilament Heavy Chain; PWd – Posterior Wall Thickness (diastolic); Q10 – Coenzyme Q10, also known as Ubiquinone; QCCR – Ubiquinol-Cytochrome c Oxidoreductase (Complex III); ROS – Reactive Oxygen Species; SARA – Scale for the Assessment and Rating of Ataxia; SD – Standard Deviation; SQR – Succinate:Quinone Oxidoreductase (Complex II); TCA cycle – Tricarboxylic Acid Cycle = Krebs Cycle; yob – Year of Birth

Supplement – Methods – Detailed Laboratory Protocols

Activities of ETC complexes NQR, SQR and COX

Blood samples were collected at the Motol University Hospital between 8-9 a.m. and were sent under controlled conditions at room temperature to the mitochondrial laboratory by 10 a.m. The results were measured at the Department of Paediatrics and Inherited Metabolic Disorders, First Medical Faculty, Charles University and General University Hospital in Prague, Prague 2, Czech Republic. All chemicals were purchased from Sigma-Aldrich (St. Louis, MO, USA). Q10 standard was obtained from TANAKA (TANAKA, Tokyo, Japan).

Samples: 9 ml of peripheral blood was collected in citrate/sodium tubes and used to isolate platelets. 2 ml of Li/heparin treated blood was used for total Q10 determination.

Platelets isolation: Platelets were isolated by differential centrifugation at room temperature (25 °C) according to Fox et al. **[39]** with minor modifications. All buffers before use were warmed to 37 °C. Platelets rich plasma was separated by centrifugation of blood sample at 130 g for 20 min (Hettich). The upper portion of plasma with platelets was collected, one drop of 0,15M citric acid was added and plasma was then centrifuged at 730 g, for 20 min. The pelleted platelets were resuspended in 5 ml buffer A (120mM NaCl, 13 mM sodium citrate, 30 mM dextrose, pH 7.0) and suspension was centrifuged at 730 g for 20 min. Pellet was resuspended in 5 ml buffer B (154 mM NaCl, 10 mM TRIS/HCl, 1 mM EDTA, pH 7.4) and centrifuged at the same condition. Final pellet of platelets was resuspended in 500 µl of buffer C (138 mM NaCl, 2.9 mM KCl, 12 mM NaHCO3, 0.36 mM NaH2PO4, 5.5 mM glucose, 1.8 mM CaCl2, 0.4 mM MgCl2, pH 7.4). Fresh cells were used for measurement of NQR and SQR; frozen aliquots of the cells stored at -80 °C were used for determination of COX and CS activity.

Activities of ETC complexes and CS: Activities of respiratory chain complexes NQR, SQR, COX, and the activity of CS serving as the control enzyme were measured spectrophotometrically according to Rustin **[40]**. All spectrophotometric measurements were performed in 1-ml cuvettes (1 cm, 37 °C) using double beam spectrophotometer Shimadzu UV-160 (Shimadzu, Japan). Each value represents the mean of two assays on each control or patient platelet sample. The ratio between activity of complex IV and CS was calculated to eliminate possible effect of changes in number of mitochondria in patient cells.

Briefly, rotenone-sensitive NQR (EC 1.6.5.3) activity was measured with 100 µg platelet protein in 1 ml of assay medium (50 mM TRIS, pH 8.1, 2.5 mg·ml-1 BSA, 50 µM decylubiquinone, 0.3 mM KCN, 0.1 mM NADH without and with 3 µM rotenone) and followed the decrease in absorbance at 340 nm due to the NADH oxidation.

SQR activity (EC 1.3.5.1) was measured by incubating 200 μg of platelet protein in 1 ml of assay medium (10 mM potassium phosphate, pH 7.8, 2 mM EDTA, 1mg·ml-1 BSA, 0.3 mM KCN, 10 mM succinate, 3 µM rotenone, 0.2 mM ATP, 80 µM 2,6-dichlorophenolindophenol (DCPIP), 1 µM antimycin, 50 µM decylubiquinone), and measuring the decrease in absorbance at 600 nm due to the reduction of DCPIP at 600 nm.

For measurement of COX and CS samples of platelets were incubated with 1.5% N-dodecyl-β-D-maltoside during 15 min at 4 °C, centrifuged at 4 °C for 10 min at 10 000g and supernatants were used for measurements. COX (EC 1.9.3.1) activity was measured by incubating 100 µg of solubilized platelet protein in 1 ml of assay medium (40 mM potassium phosphate, pH 7.0, 1 mg·ml-1 BSA, 25 µM reduced cytochrome c) by following the oxidation of cytochrome c at 550 nm.

CS (EC 2.3.3.1) was measured according to Srere **[41]** in assay medium consisted of 100 mM TRIS/HCl pH 8.1, 0.1 mM 5,5´-dithiobis-(2-nitrobenzoic) acid, 100 µg solubilized platelet protein, 0.5 mM acetyl coenzyme A. In the first step, the background at 412 nm was measured during 1 min. Reaction was started by addition of 0.5 mM oxalacetic acid. Resulted activity was calculated after subtraction of background.

Q10 levels assessment: Total Q10 content in plasma was determined according to Mosca **[42]**. 200 mL of plasma was vortexed with the addition of 50 µL 1,4-benzoquinone (2 mg/mL; Sigma-Aldrich, Saint Louis, MO, USA) and left for 10 min at room temperature. Then, 1 mL of propan-1-ol was added and the mixture was vortexed properly. Samples were centrifuged (26,000× g, 20 min, 4 ◦C) and 50 uL of the acquired supernatant was used for HPLC analysis (HPLC 20 prominence system, Shimadzu) using the Supelcosil LC 18 column (Supelco) 25 cm x 0.46 cm i.d. 5 cm, precolumn LC 18S, 2 cm (Supelco). An in-line filter A-701 (Upchurch Scientific, Inc., Oak Harbor, WA) was placed between the injector and the precolumn eluted by ethanol/methanol (70/30 v/v) mixture at a flow rate of 1 ml/min. UV detection was performed at 275 nm. Results were expressed as µg of Q10 per mL.

Protein content: Protein content was determined by Lowry **[43]**.

Neurofilament Light Chain (NFL) Assessment via ELISA: Blood samples were collected at the Motol University Hospital between 8-9 a.m. and were sent at room temperature to the immunological and CSF laboratory of the same hospital within 20 minutes from the blood draw. The results were measured at the Department of Immunology, Second Faculty of Medicine, Charles University, Motol University Hospital, Prague 5, Czech Republic. For the measurement of neurofilament light chain (NFL) levels in blood samples, we employed the NF-light™ Serum ELISA RUO kit **[44]**, sourced from BioTech a.s, Prague, Czech Republic. The kit, with a catalogue number of 20-8002 labelled for research use only (RUO), incorporates a 96-well strip plate, and utilizes a colorimetric ELISA (Enzyme-Linked Immunosorbent Assay) technique with a TMB substrate.

N-terminal Pro-Brain Natriuretic Peptide (NT-proBNP): the NT-proBNP test is measured in units of ng/L. The specimen required for this test is serum, which remains stable at a temperature range of 4 - 8 °C for up to 6 days according to the specifications outlined by Guder. The principal methodology employed in this test is the Chemiluminescent Immunoassay (CMIA). For sample collection, the VACUETTE system is used, specifically a tube with a hemocoagulation accelerator, indicated by a red cap. The biological half-life of NT-proBNP is approximately 2 hours, and its biological variability has an intra-individual variability of %CVw = 10.0 as stated by Westgard. The transportation and storage of the sample require immediate transfer to the laboratory following collection. Analytical interferences that can affect the test results include hemolysis starting from a hemoglobin level of 10.0 g/L, icterus from a bilirubin level of 1026 µmol/L, and chylomicronemia from triacylglycerols level of 34.2 mmol/L. The test's reference ranges vary with age and are applicable to both genders. The reference ranges for the NT-proBNP test are stratified by age, providing specific values for each group: from 0 to 2 days, the range is 321 - 11987 ng/L; for ages 3 to 11 days, it's 263 - 5918 ng/L; from 31 to 365 days, the range narrows to 37.0 - 646 ng/L; for children aged 1 to 2 years, it's 39.0 - 413 ng/L; between 2 and 6 years, the range is 23.0 - 289 ng/L; for ages 6 to 14 years, it further decreases to 10.0 - 157 ng/L; in teenagers from 14 to 18 years, it is 6.0 - 158 ng/L; adults aged 18 to 75 years have a range of 20.0 - 125 ng/L; and finally, for the elderly between 75 and 115 years, the range is 20.0 - 450 ng/L. These ranges were established based on studies by Nir et al., as published in Pediatric Cardiology in 2009. A key calculation in this test is the conversion of units from pmol/L to ng/L, using a factor of 8.457. This test is integral to clinical practices, particularly in the areas of cardiovascular and renal health **[45-46]**.

Myoglobin: the myoglobin test is measured in units of μg/L. The required specimen for this assay is serum, which maintains its stability at a temperature range of 4 - 8 °C for up to 7 days, as noted by Guder. The principal technique employed for this test is the Chemiluminescent Immunoassay (CMIA). For the collection of the sample, the VACUETTE system is utilized, specifically a tube with a hemocoagulation accelerator, recognizable by its red cap. The biological half-life of Myoglobin is notably short, approximately 15 minutes, and the biological variability of this biomarker presents an intra-individual variability of %CVw = 13.9, according to Westgard. The sample, once collected, necessitates immediate transport to the laboratory to preserve its integrity. The test is susceptible to analytical interferences such as hemolysis, which becomes significant from a hemoglobin concentration of 5.0 g/L, icterus starting from a bilirubin level of 342 μmol/L, and chylomicronemia from triacylglycerols level of 11.4 mmol/L. Reference ranges for the Myoglobin test are established for both genders and vary across different age groups. For instance, from 0 to 6 months, the range is 13 – 50 μg/L, extending to 19 – 92 μg/L for men and 12 – 76 μg/L for women aged between 18 and 115 years. These reference ranges were derived from studies conducted by Zima et al. and Rödöö, P., Ridefelt, P., as published in Scand. J. of Clin & Lab Investigation in 2013. A crucial aspect of this test is the conversion of units from nmol/L to μg/L, using a factor of 0.0171 **[45-46]**.

Supplement – Figures and Datasets

**Figure X1 Chart Matrix X1A-X1C: Effect of Succinate:quinone Oxidoreductase versus Citrate Synthase Activities in FA Patients and Healthy Controls**

**Chart X1A: Scatterplot and Density Distribution of Succinate:quinone Oxidoreductase versus Citrate Synthase Activities in FA Patients and Healthy Controls**

This scatterplot examines the relationship between succinate:quinone oxidoreductase (SQR, Complex II) activity in (nmol/min*mg protein) on the x-axis and citrate synthase (CS) activity in (nmol/min*mg protein) on the y-axis within the Friedreich's Ataxia (FA) patient cohort (P) and healthy control (HC) group. Data points are color-coded, with FA patients represented in orange and healthy controls in green. The distribution of SQR activity is shown along the bottom x-axis, while the distribution of CS activity is along the left y-axis. Kernel density estimation curves adjacent to each axis demonstrate the distribution density of activities for SQR and CS. Notably, a comparison within the data suggests a tendency for FA patients to exhibit reduced SQR activity in relation to CS activity when compared to healthy controls.

**Chart X1B: Linear Regression Analysis of Succinate:quinone Oxidoreductase vs. Citrate Synthase Activities in FA Patients**

This chart showcases a linear regression analysis where citrate synthase (CS) activity in (nmol/min*mg protein) is plotted on the y-axis as a function of succinate:quinone oxidoreductase (SQR, Complex II) activity in (nmol/min*mg protein) on the x-axis within the Friedreich's Ataxia (FA) patient cohort (P), represented by orange points. The linear regression line is shown in orange, with a shaded area denoting the 95% confidence interval (CI) that estimates the precision of the regression fit. The histogram at the top reflects the distribution of SQR activity, while the boxplot to the right displays the distribution of CS activity, both with kernel density estimations to visualize the distribution density. The regression suggests a relationship between SQR and CS activities among FA patients, as indicated by the upward trend in the plot.

**Chart X1C: Linear Regression Analysis of Succinate:quinone Oxidoreductase vs. Citrate Synthase Activities in Healthy Controls**

This chart illustrates a linear regression analysis depicting citrate synthase (CS) activity in (nmol/min*mg protein) as the dependent variable on the y-axis against succinate:quinone oxidoreductase (SQR, Complex II) activity in (nmol/min*mg protein) on the x-axis among healthy controls (HC), marked with green points. The fitted regression line is displayed in green, with a surrounding shaded area representing the 95% confidence interval (CI), which conveys the reliability of the estimated relationship. The histogram above shows the distribution of SQR activity, and the boxplot to the right delineates the spread of CS activity. Both are accompanied by kernel density plots that outline the distribution density of each activity. The analysis demonstrates a positive trend, suggesting an association between SQR and CS activities in the healthy population.

**Table X1A – supplement: A comprehensive profile of the Healthy Control (HC) cohort**

A comprehensive profile of the HC cohort including demographic information such as age and gender, and serum neurofilament light chain levels (NFL).

P – FA patient; HC – healthy control; M – male; F – female; NFL – neurofilament light chain level; NA – not available.

|  | **P_HC** | **age at examination** | **Gender (1=M, 2=F)** | **NFL (pg/ml)** |
| --- | --- | --- | --- | --- |
| **106** | HC | 13 | 1 | NA |
| **80** | HC | 14 | 1 | NA |
| **49** | HC | 16 | 1 | NA |
| **42** | HC | 18 | 2 | NA |
| **23** | HC | 23 | 1 | 7.80 |
| **96** | HC | 25 | 1 | 7.89 |
| **91** | HC | 24 | 2 | 4.12 |
| **8** | HC | 26 | 2 | 4.19 |
| **55** | HC | 26 | 1 | 4.50 |
| **90** | HC | 27 | 1 | 5.71 |
| **71** | HC | 31 | 2 | 4.26 |
| **53** | HC | 36 | 1 | 5.91 |
| **88** | HC | 37 | 2 | 9.11 |
| **12** | HC | 40 | 2 | 1.90 |
| **4** | HC | 42 | 2 | 8.10 |
| **97** | HC | 50 | 1 | 9.84 |
| **116** | HC | 59 | 2 | NA |
|  | **HC N** | **17** | **17** | **12** |
|  | **HC mean** | **30** |  | **6.11** |
|  | **HC max** | **59** |  | **9.84** |
|  | **HC min** | **13** |  | **1.90** |
|  | **HC SD** | **13** |  | **2.42** |

**Table X1B – supplement: A comprehensive profile of the FA patient (P) cohort**

Our study presents a comprehensive profile of the FA patient (P) cohort, encompassing demographic details such as age and gender, along with a variety of clinical variables sourced from the EFACTS database. This includes age at FA onset, duration of the disease, gene variation (the length of GAA expansions or point mutations), disability status, severity index, and the age at which FA patients required permanent walking aids and became wheelchair dependent. Additionally, the data encompasses ADL and SARA scores, with a calculated average annual worsening for each, based on the disease duration. Our analysis also includes results from the MoCA test for cognitive assessment and NFL levels, which were measured using ELISA.

P – FA patient; HC – healthy control; M – male; F – female; GAA1/GAA2 – shorter/longer allele with Guanine-Adenine-Adenine repeats; ADL – Activities of Daily Living; SARA – Scale for Assessment and Rating of Ataxia; AVG ADL – Average Activities of Daily Living per year of disease duration; AVG SARA – Average Scale for the Assessment and Rating of Ataxia per year of disease duration; MoCA – Montreal Cognitive Assessment; NFL – neurofilament light chain level; NA – not available.

|  | **P_HC** | **age at examination** | **Gender (1=M, 2=F)** | **onset (age)** | **disease duration** | **GAA1** | **GAA2** | **point mutation** | **disability** | **severity index** | **when perm wheelchair** | **when perm walking aid** | **ADL sum** | **SARA sum** | **AVG ADL** | **AVG SARA** | **MoCA total** | **NFL (pg/ml)** |
| --- | --- | --- | --- | --- | --- | --- | --- | --- | --- | --- | --- | --- | --- | --- | --- | --- | --- | --- |
| **99** | P | 7 | 2 | 1 | 7 | 1160 | 1330 | none | 3 | NA | NA | NA | 17 | 9 | 2.42 | 1.28 | NA | 50.91 |
| **86** | P | 12 | 1 | 3 | 9 | 1000 | 1100 | none | 6 | NA | 9 | 5 | 20 | 31 | 2.12 | 3.29 | NA | 48.82 |
| **84** | P | 14 | 2 | 11 | 4 | 760 | 1000 | none | 3 | 1.00 | NA | NA | 4 | 10 | 1.06 | 2.66 | 29 | 19.52 |
| **102** | P | 15 | 1 | 12 | 4 | 660 | 830 | none | 3 | 1.00 | NA | NA | 4 | 8 | 0.98 | 1.97 | 24 | 24.06 |
| **93** | P | 16 | 2 | 11 | 5 | 660 | 930 | none | 4 | 0.80 | NA | 15 | 9 | 12 | 1.67 | 2.23 | 24 | 12.77 |
| **60** | P | 18 | 2 | 6 | 13 | 1000 | 1100 | none | 6 | 0.50 | 16 | 14 | 17 | 26.5 | 1.32 | 2.06 | 27 | 12.48 |
| **104** | P | 19 | 2 | 15 | 4 | 560 | 1000 | none | 2 | 0.50 | NA | NA | 2 | 10 | 0.50 | 2.51 | 30 | 18.66 |
| **66** | P | 20 | 2 | 4 | 17 | 760 | 830 | none | 6 | 0.38 | 12 | 7 | 24 | 32 | 1.43 | 1.91 | NA | 28.98 |
| **22** | P | 21 | 1 | 13 | 9 | 660 | 830 | none | 5 | 0.63 | 19 | 17 | 9 | 17 | 1.04 | 1.97 | 29 | 14.22 |
| **76** | P | 23 | 1 | 11 | 13 | 430 | 1000 | none | 6 | 0.50 | 23 | 22 | 12 | 13.5 | 0.93 | 1.04 | 28 | 19.48 |
| **68** | P | 23 | 1 | 12 | 12 | 530 | 1000 | none | 5 | 0.45 | 23 | 19 | 15 | 19.5 | 1.26 | 1.63 | 27 | 10.50 |
| **72** | P | 23 | 1 | 12 | 11 | 830 | 1000 | none | 6 | 0.55 | 20 | 18 | 19 | 24 | 1.73 | 2.18 | 28 | NA |
| **62** | P | 24 | 2 | 9 | 16 | 660 | 1160 | none | 6 | 0.40 | 22 | 18 | 20 | 25.5 | 1.24 | 1.59 | NA | 10.39 |
| **65** | P | 29 | 1 | 18 | 12 | 230 | 400 | none | 3 | 0.27 | NA | NA | 6 | 8 | 0.49 | 0.66 | 27 | 16.68 |
| **15** | P | 29 | 1 | 7 | 23 | 660 | 830 | none | 7 | 0.32 | 15 | 15 | 26 | 34 | 1.13 | 1.48 | 27 | 21.77 |
| **82** | P | 29 | 1 | 17 | 12 | 230 | 830 | none | 3 | NA | NA | NA | 12 | 14 | 0.98 | 1.15 | 27 | 15.12 |
| **25** | P | 30 | 1 | 1 | 30 | 660 | 830 | none | 6 | 0.21 | 17 | 15 | 23 | 34 | 0.76 | 1.13 | 27 | 23.61 |
| **5** | P | 31 | 2 | 3 | 29 | 1100 | 1160 | none | 7 | 0.25 | 12 | 10 | 25 | 37 | 0.88 | 1.30 | 9 | 16.75 |
| **35** | P | 32 | 1 | 7 | 25 | 930 | 1000 | none | 6 | 0.24 | 21 | 20 | 16 | 28 | 0.64 | 1.12 | 28 | 11.06 |
| **10** | P | 32 | 1 | 5 | 28 | 830 | NA | C165+1 G>C | 6 | 0.22 | 15 | 13 | 20 | 25 | 0.71 | 0.89 | NA | 10.51 |
| **27** | P | 33 | 1 | 12 | 22 | 430 | 830 | none | 6 | 0.29 | 21 | 16 | 22 | 31 | 1.01 | 1.42 | 23 | 16.14 |
| **48** | P | 34 | 2 | 16 | 19 | 630 | 1000 | none | 6 | 0.33 | 31 | 19 | 16 | 23.5 | 0.85 | 1.24 | 25 | 14.36 |
| **78** | P | 35 | 1 | 21 | 14 | 90 | 330 | none | 3 | 0.21 | NA | NA | 7 | 7 | 0.49 | 0.49 | 27 | 11.82 |
| **34** | P | 36 | 1 | 16 | 20 | 830 | 1000 | none | 6 | 0.30 | 24 | 23 | 19 | 29 | 0.95 | 1.45 | 29 | 20.97 |
| **18** | P | 36 | 2 | 10 | 27 | 500 | 1160 | none | 6 | 0.23 | 21 | 16 | 24 | 35 | 0.90 | 1.32 | 23 | 15.88 |
| **51** | P | 38 | 2 | 11 | 28 | 630 | 1000 | none | 6 | 0.22 | 33 | 26 | 20 | 24.5 | 0.72 | 0.88 | 30 | 10.19 |
| **56** | P | 42 | 1 | 19 | 23 | 500 | 560 | none | 4 | 0.17 | NA | NA | 11 | 11 | 0.47 | 0.47 | 25 | 20.20 |
| **37** | P | 44 | 1 | 12 | 32 | 200 | 300 | none | 5 | 0.16 | NA | 39 | 13 | 18 | 0.41 | 0.57 | 28 | 17.92 |
| **3** | P | 46 | 2 | 19 | 27 | 830 | 1000 | none | 6 | 0.22 | 25 | 22 | 27 | 40 | 0.99 | 1.47 | NA | 0.74 |
| **32** | P | 47 | 1 | 8 | 40 | 500 | 1000 | none | 6 | 0.15 | 46 | 40 | 12 | 21 | 0.30 | 0.53 | 26 | 6.76 |
| **1** | P | 47 | 2 | 10 | 37 | 830 | 930 | none | 6 | 0.16 | 25 | 20 | 25 | 36.5 | 0.67 | 0.97 | 18 | 25.12 |
| **20** | P | 49 | 1 | 12 | 37 | 830 | 930 | none | 6 | 0.16 | 30 | 28 | 20 | 29.5 | 0.54 | 0.79 | 27 | 6.52 |
| **13** | P | 53 | 2 | 18 | 35 | 760 | 830 | none | 6 | 0.17 | 39 | 35 | 14 | 31 | 0.40 | 0.88 | 25 | 5.94 |
| **29** | P | 55 | 2 | 33 | 22 | 130 | 150 | none | 5 | 0.23 | NA | 46 | 12 | 13 | 0.54 | 0.59 | 27 | 23.44 |
|  | **P N** | **34** | **34** | **34** | **34** | **34** | **33** | **1** | **34** | **31** | **23** | **26** | **34** | **34** | **34** | **34** | **28** | **33** |
|  | **P mean** | **31** |  | **12** | **20** | **647** | **884** | **NA** | **5** | **0.36** | **23** | **21** | **16** | **22.6** | **0.96** | **1.39** | **26** | **17.65** |
|  | **P max** | **55** |  | **33** | **40** | **1160** | **1330** | **NA** | **7** | **1.00** | **46** | **46** | **27** | **40.0** | **2.42** | **3.29** | **30** | **50.91** |
|  | **P min** | **7** |  | **1** | **4** | **90** | **150** | **NA** | **2** | **0.15** | **9** | **5** | **2** | **7.0** | **0.30** | **0.47** | **9** | **0.74** |
|  | **P SD** | **12** |  | **6** | **10** | **267** | **264** | **NA** | **1** | **0.23** | **9** | **10** | **7** | **10** | **0.49** | **0.68** | **4** | **10.39** |

**Table X2A – supplement: A comprehensive analysis of enzymatic activities in isolated platelets and Q10 levels in plasma for the HC cohort**

A comprehensive analysis of enzymatic activities and biochemical levels for the healthy control (HC) cohort – no one of this cohort reported Q10 supplementation.

P – FA patient; HC – healthy control; NQR – ETC’s Complex I activity; SQR – ETC’s Complex II activity; Q10 – Coenzyme Q10 (ubiquinone) level; COX – ETC’s Complex IV activity; COX/SC – COX/CS ratio; NA – not available.

|  | **P_HC** | **NQR**  **(nmol/min*mg prot)** | **SQR**  **(nmol/min*mg prot)** | **Q10**  **(µg/ml)** | **COX**  **(nmol/min*mg prot)** | **CS**  **(nmol/min*mg prot)** | **COX/CS** |
| --- | --- | --- | --- | --- | --- | --- | --- |
| **106** | HC | 37.30 | 10.80 | 0.41 | 16.03 | 59.08 | 0.27 |
| **80** | HC | 30.83 | 14.75 | 0.73 | 23.74 | 78.44 | 0.30 |
| **49** | HC | 56.40 | 8.50 | 0.40 | 21.26 | 72.30 | 0.29 |
| **42** | HC | 56.80 | 15.60 | 0.51 | 23.92 | 84.25 | 0.28 |
| **23** | HC | 45.70 | 27.40 | 0.54 | 29.12 | 90.99 | 0.32 |
| **96** | HC | 115.30 | 25.82 | 0.36 | 24.64 | 73.81 | 0.33 |
| **91** | HC | 54.00 | 33.88 | 0.57 | 28.61 | 68.89 | 0.42 |
| **8** | HC | 95.00 | 37.97 | 0.61 | 25.56 | 77.79 | 0.33 |
| **55** | HC | 132.00 | 18.52 | 0.65 | 23.96 | 69.07 | 0.35 |
| **90** | HC | 31.20 | 17.40 | 0.57 | 19.04 | 69.83 | 0.27 |
| **71** | HC | 43.00 | 23.67 | 0.63 | 22.05 | 63.88 | 0.35 |
| **53** | HC | 54.16 | 20.99 | 1.07 | 11.31 | 71.18 | 0.16 |
| **88** | HC | 53.17 | 19.30 | 0.79 | 31.03 | 79.62 | 0.39 |
| **12** | HC | 85.66 | 18.48 | 1.21 | 28.72 | 74.20 | 0.39 |
| **4** | HC | 70.60 | 16.49 | 0.37 | 22.28 | 64.49 | 0.35 |
| **97** | HC | 21.70 | 21.70 | 1.04 | 19.15 | 74.45 | 0.26 |
| **116** | HC | 68.32 | NA | NA | 9.75 | 72.59 | 0.13 |
|  | **HC N** | **17** | **16** | **16** | **17** | **17** | **17** |
|  | **HC mean** | **61.83** | **20.70** | **0.65** | **22.36** | **73.23** | **0.31** |
|  | **HC max** | **132.00** | **37.97** | **1.21** | **31.03** | **90.99** | **0.42** |
|  | **HC min** | **21.70** | **8.50** | **0.36** | **9.75** | **59.08** | **0.13** |
|  | **HC SD** | **30.15** | **7.75** | **0.26** | **5.95** | **7.69** | **0.08** |

**Table X2B – supplement: A comprehensive analysis of enzymatic activities in isolated platelets and Q10 levels in plasma for the FA patient (P) cohort**

A comprehensive analysis of enzymatic activities and biochemical levels for the FA patient (P) cohort**.**

P – FA patient; HC – healthy control; NQR – ETC’s Complex I activity; SQR – ETC’s Complex II activity; Q10 – Coenzyme Q10 (ubiquinone) level; COX – ETC’s Complex IV activity; COX/CS – COX/CS ratio; NA – not available; prot – protein.

|  | **P_HC** | **NQR**  **(nmol/min*mg prot)** | **SQR**  **(nmol/min*mg prot)** | **Q10**  **(µg/ml)** | **COX**  **(nmol/min*mg prot)** | **CS**  **(nmol/min*mg prot)** | **COX/CS** |
| --- | --- | --- | --- | --- | --- | --- | --- |
| **99** | P | 47.30 | 15.30 | 1.15 | 13.31 | 64.09 | 0.21 |
| **86** | P | 40.86 | 13.62 | 0.70 | 14.58 | 76.36 | 0.19 |
| **84** | P | 19.00 | 6.00 | 0.49 | 14.12 | 58.02 | 0.24 |
| **102** | P | 31.03 | 14.92 | 0.99^ | 12.12 | 93.47 | 0.13 |
| **93** | P | 45.60 | 15.02 | 0.89 | 10.38 | 75.27 | 0.14 |
| **60** | P | 34.34 | 13.17 | 0.78 | 19.63 | 68.64 | 0.29 |
| **104** | P | NA | NA | 0.33 | NA | NA | NA |
| **66** | P | 30.82 | 10.58 | 0.38 | 12.42 | 58.49 | 0.21 |
| **22** | P | 52.35 | 11.28 | 0.47 | 8.55 | 77.51 | 0.11 |
| **76** | P | 69.60 | 13.10 | 0.78 | 8.24 | 80.69 | 0.10 |
| **68** | P | 67.10 | 9.90 | 0.85 | 10.80 | 86.26 | 0.13 |
| **72** | P | 45.50 | 14.40 | 0.99 | 10.97 | 79.04 | 0.14 |
| **62** | P | 35.52 | 10.17 | 0.76 | 7.32 | 35.75 | 0.20 |
| **65** | P | 58.45 | 7.89 | 0.37 | 6.38 | 73.90 | 0.09 |
| **15** | P | 47.51 | 13.75 | 0.66^ | 10.74 | 76.33 | 0.14 |
| **82** | P | NA | 22.26 | 0.62 | 10.78 | 79.32 | 0.14 |
| **25** | P | 46.12 | 13.81 | 0.87 | 9.15 | 75.76 | 0.12 |
| **5** | P | 31.04 | 14.86 | 2.28^ | 13.44 | 67.05 | 0.20 |
| **35** | P | 65.60 | 8.00 | 0.72 | 9.18 | 39.09 | 0.23 |
| **10** | P | 36.10 | 7.60 | 1.58^ | 11.40 | 81.36 | 0.14 |
| **27** | P | NA | 18.46 | 0.66 | 10.03 | 88.66 | 0.11 |
| **48** | P | 22.95 | 11.69 | 0.81 | 8.36 | 70.19 | 0.12 |
| **78** | P | 71.72 | 16.26 | 0.76 | 17.67 | 76.20 | 0.23 |
| **34** | P | 75.20 | 15.10 | 0.88 | 24.37 | 103.62 | 0.24 |
| **18** | P | 47.31 | 11.52 | 1.49 | 19.30 | 64.97 | 0.30 |
| **51** | P | 53.93 | 9.05 | 0.36 | 8.75 | 70.27 | 0.13 |
| **56** | P | 55.90 | 11.30 | 1.34 | 26.48 | 72.02 | 0.37 |
| **37** | P | 39.20 | 9.10 | 0.62 | 18.29 | 61.54 | 0.30 |
| **3** | P | NA | NA | 1.06 | NA | NA | NA |
| **32** | P | NA | NA | 0.92 | NA | NA | NA |
| **1** | P | NA | NA | 1.00^ | NA | NA | NA |
| **20** | P | NA | NA | 1.28 | NA | NA | NA |
| **13** | P | NA | NA | 0.78 | NA | NA | NA |
| **29** | P | 38.10 | 13.40 | 0.65 | 8.29 | 69.82 | 0.12 |
|  | **P N** | **26** | **28** | **34** | **28** | **28** | **28** |
|  | **P mean** | **46.47** | **12.55** | **0.86** | **12.68** | **72.27** | **0.18** |
|  | **P max** | **75.20** | **22.26** | **2.28** | **26.48** | **103.62** | **0.37** |
|  | **P min** | **19.00** | **6.00** | **0.33** | **6.38** | **35.75** | **0.09** |
|  | **P SD** | **15.07** | **3.54** | **0.39** | **5.07** | **14.09** | **0.07** |

^ FA patients (P) supplementing Q10 on the time of examination are marked with a caret

**Table X3 – supplement: Cardiac and Muscle Biomarkers in FA Patients and Healthy Controls**

In this table, we present a comparative analysis of cardiac and muscle biomarkers between FA patients (P) and healthy controls. The data show a higher average level of the cardiomarker NT-proBNP, which is indicative of atrial dilation in heart failure. It's noteworthy to mention that cardiopathy linked with FA is a common finding. Although severe pathological levels for NT-proBNP often exceed the three thousand mark, the data from FA patients (P) did not consistently reach such elevations suggesting that this is more likely chronic minor damage of cardiomyocytes in usually hypertrophic FA heart. Most of the patients with higher levels are presenting with worse cardiac issues, typically arrythmias.

Conversely, we observed a trend toward reduced myoglobin levels in FA patients (P). Such a decrease could be attributed to muscle atrophy, resulting in reduced lean muscle mass and subsequently, decreased myoglobin presence in the bloodstream. Alternatively, it may also signify damage to cardiomyocytes. It's important to note that we were not able to separate myoglobin exclusive to cardiomyocytes and muscle cells.

**Table X3A – supplement: Cardiac and Muscle Biomarkers in HC group**

P – FA patient; HC – healthy control; NT-proBNP – N-Terminal pro b-type Natriuretic Peptide level, a cardiac marker of atrial dilation in heart failure; NA – not available

|  | **P_HC** | **Myoglobin (µg/L)** | **NT-proBNP (ng/L)**  (a minimal detection limit is 35 ng/L) |
| --- | --- | --- | --- |
| **96** | HC | 58.4 | 55.5 |
| **49** | HC | NA | NA |
| **55** | HC | 79.3 | 35.0 |
| **90** | HC | 42.7 | 38.2 |
| **23** | HC | 50.3 | 35.0 |
| **80** | HC | NA | NA |
| **71** | HC | 44.9 | 35.0 |
| **91** | HC | 28.6 | 47.7 |
| **106** | HC | NA | NA |
| **4** | HC | 44.7 | 82.2 |
| **42** | HC | 25.3 | 35.0 |
| **8** | HC | 28.4 | 45.4 |
| **88** | HC | 30.5 | 132.7 |
| **53** | HC | 46.9 | 35.0 |
| **12** | HC | 33.9 | 58.1 |
| **97** | HC | 36.5 | 35.0 |
| **HC N** | | **13** | **13** |
| **HC mean** | | **42.34** | **51.52** |
| **HC max** | | **79.30** | **132.70** |
| **HC min** | | **25.30** | **35.00** |
| **HC SD** | | **14.83** | **28.07** |

**Table X3B – supplement: Cardiac and Muscle Biomarkers in FA patient (P) group**

P – FA patient; HC – healthy control; NT-proBNP – N-Terminal pro b-type Natriuretic Peptide level, a cardiac marker of atrial dilation in heart failure; IVS – intraventricular septum; PWd – posterior wall thickness (diastolic); NA – not available

|  | **P_HC** | **Myoglobin (µg/L)** | **NT-proBNP (ng/L)**  (a minimal detection limit is 35 ng/l) | **palpitations age** | **chest pain age** | **dyspnoe age** | **IVS thickness mm** | **PWD thickness mm** | | **ejection fraction %** |
| --- | --- | --- | --- | --- | --- | --- | --- | --- | --- | --- |
| **78** | P | 31.3 | 35.0 | NA | 33 | 30 | 12 | 11 | | 85 |
| **29** | P | 41.7 | 40.2 | NA | NA | NA | 9 | 9 | | 65 |
| **37** | P | 51.4 | 35.9 | NA | NA | NA | 7 | 7 | | 65 |
| **82** | P | 36.6 | 35.0 | 23 | 23 | NA | 11 | 11 | | 70 |
| **65** | P | 36.3 | 36.1 | NA | NA | NA | 11 | 10 | | 60 |
| **76** | P | 59.5 | 35.0 | NA | NA | NA | 11 | 9 | | 65 |
| **27** | P | 66.5 | 35.0 | NA | NA | NA | 12 | 12 | | 64 |
| **56** | P | 84.7 | 48.9 | NA | NA | NA | 8 | 9 | | 65 |
| **32** | P | 59.0 | 35.0 | 45 | NA | 45 | 12 | 11 | | 70 |
| **18** | P | 13.5 | 120.5 | NA | NA | 36 | 15 | 11 | | 65 |
| **68** | P | 37.4 | 35.0 | NA | NA | 18 | 14 | 10 | | 65 |
| **104** | P | 21.6 | 35.0 | NA | NA | NA | 7 | 22 | | 65 |
| **51** | P | 26.6 | 39.4 | NA | NA | 18 | NA | NA | | NA |
| **48** | P | 19.4 | 259.0 | NA | NA | NA | NA | NA | | 61 |
| **15** | P | 41.1 | 69.1 | NA | NA | 13 | 16 | 11 | | 65 |
| **22** | P | 33.6 | 813.0 | 20 | NA | NA | 17 | 15 | | 70 |
| **25** | P | 42.0 | 35.0 | NA | 25 | 24 | 7 | 8 | | 60 |
| **93** | P | 48.1 | 305.9 | NA | 14 | 14 | 12,8 | 13,1 | | 59 |
| **62** | P | 23.6 | 47.3 | 18 | 21 | 24 | 11 | 10 | | 60 |
| **102** | P | 42.8 | 35.0 | NA | NA | NA | 12 | 11 | | 70 |
| **66** | P | 19.1 | 114.9 | NA | NA | NA | 11 | 10 | | 55 |
| **13** | P | 50.1 | 87.9 | NA | NA | NA | 10 | 8 | | 65 |
| **84** | P | 31.4 | 35.0 | 10 | 10 | 10 | 9,3 | 8,6 | | 64 |
| **10** | P | 39.6 | 35.0 | 11 | NA | 31 | 15,5 | 10 | | 60 |
| **72** | P | 39.0 | 35.0 | 19 | 19 | NA | 14 | 12 | | 60 |
| **34** | P | 59.2 | 35.0 | NA | NA | 33 | 13 | 9 | | 65 |
| **20** | P | 74.0 | 377.7 | NA | NA | 9 | 28 | 13 | | 65 |
| **1** | P | 25.7 | 153.5 | 42 | 42 | 42 | NA | NA | | NA |
| **3** | P | 47.7 | 40.5 | NA | NA | NA | NA | NA | | NA |
| **35** | P | 21.7 | 26.4 | 30 | 30 | NA | 11 | 10 | | 67 |
| **60** | P | 25.4 | 35.0 | 6 | 6 | NA | 30 | 15 | | 90 |
| **86** | P | 28.4 | 91.3 | NA | NA | 8 | 13,3 | 10,7 | | 63 |
| **5** | P | 25.6 | 348.4 | NA | NA | 25 | 12 | 10 | | 50 |
| **99** | P | 47.4 | 104.1 | NA | NA | NA | 9,4 | 8,4 | | 75 |
| **P N** | | **34** | **34** | **10** | **10** | **16** | **30** | **30** | | **31** |
| **P mean** | | **39.74** | **107.35** | **22.4** | **22.3** | **23.8** | **12.7** | **10.8** | **65.4** | |
| **P max** | | **84.70** | **813.00** | **45** | **42** | **45** | **30** | **22** | **90** | |
| **P min** | | **13.50** | **26.40** | **6** | **6** | **8** | **7** | **7** | **50** | |
| **P SD** | | **16.57** | **156.35** | **13.1** | **10.9** | **11.7** | **5.1** | **2.8** | **7.6** | |

**Table X4 – supplement: Regression Model for Neurofilament Light Chain Levels and FA Patient Age at examination**

Table X4 below summarizes the findings from the linear regression analysis, providing coefficient estimates for the intercept and age at baseline, along with their respective standard errors, t-values, and p-values. It quantifies the effect of patient age on neurofilament light chain (NFL) levels, indicating a significant inverse relationship where older patients showed approximately 0.4 pg/ml lower NFL levels per year of age.

| **Endogenous Variable** | **R² (r)** | **N** | **Regressor** | **Coefficient** | **Std. Error** | **t-value** | **p-value** |
| --- | --- | --- | --- | --- | --- | --- | --- |
| **NFL pg/ml** | 0.2559 | 33 | Intercept | 30.8309 | 4.3688 | 7.057 | **< 0.001** |
|  |  |  | **Age at examination in years** | -0.4351 | 0.1312 | -3.318 | **< 0.003** |
